# Simultaneous Pumping and Mixing of Biological Fluids in a Double-Array Electrothermal Microfluidic Device

**DOI:** 10.3390/mi10020092

**Published:** 2019-01-28

**Authors:** Alinaghi Salari, Colin Dalton

**Affiliations:** 1 Biomedical Engineering Graduate Program, Ryerson University, Toronto, ON M5B 2K3, Canada; 2 Institute for Biomedical Engineering, Science and Technology (iBEST), St. Michael’s Hospital, Toronto, ON M5B 1T8, Canada; 3 Keenan Research Centre, St. Michael’s Hospital, Toronto, ON M5B 1T8, Canada; 4 Electrical and Computer Engineering Department, University of Calgary, Calgary, AB T2N 1N4, Canada; 5 Biomedical Engineering Graduate Program, University of Calgary, Calgary, AB T2N 1N4, Canada

**Keywords:** electrokinetics, microfluidics, electrothermal, micropump, micromixing, biofluid, microelectrode array

## Abstract

Transport and mixing of minute amounts of biological fluids are significantly important in lab-on-a-chip devices. It has been shown that the electrothermal technique is a suitable candidate for applications involving high-conductivity biofluids, such as blood, saliva, and urine. Here, we introduce a double-array AC electrothermal (ACET) device consisting of two opposing microelectrode arrays, which can be used for simultaneous mixing and pumping. First, in a 2D simulation, an optimum electrode-pair configuration capable of achieving fast transverse mixing at a microfluidic channel cross-section is identified by comparing different electrode geometries. The results show that by adjusting the applied voltage pattern and position of the asymmetrical microelectrodes in the two arrays, due to the resultant circular flow streamlines, the time it takes for the analytes to be convected across the channel cross-section is reduced by 95% compared to a diffusion-only-based transport regime, and by 80% compared to a conventional two-layer ACET device. Using a 3D simulation, the fluid transport (pumping and mixing) capabilities of such an electrode pair placed at different angles longitudinally relative to the channel was studied. It was found that an asymmetrical electrode configuration placed at an angle in the range of 30°≤θ≤45° can significantly increase transversal mixing efficiency while generating strong longitudinal net flow. These findings are of interest for lab-on-a-chip applications, especially for biosensors and immunoassays, where mixing analyte solutions while simultaneously moving them through a microchannel can greatly enhance the sensing efficiency.

## 1. Introduction

Microscale fluid manipulation techniques have received much attention over the last decade due to the wide range of applications in various research fields including medicine, chemistry, and biology [1,2,3]. One major challenge in microfluidic devices is the manipulation of fluids and droplets effectively in such scales. Due to the laminar flow regime (i.e., low Reynolds number) in microfluidic devices, mixing of species is also difficult and unless an active mixing strategy is employed, passive diffusion is the only mechanism that causes the fluid to mix. For many applications, diffusion is considered too slow [4,5,6].

Many active strategies for pumping and mixing fluids have been reported in the literature [7,8]. In mechanical micropumps, physical actuators or mechanical parts (e.g., diaphragm and check valves) are utilized in order to generate a pressure difference across a liquid bulk. Mechanical micropumps can be actuated in multiple ways: piezoelectrically, pneumatically, electrostatically, and electromagnetically [7,8]. However, the main drawback of these micropumps is related to the nature of the diaphragm, which generates an undesirable pulsed flow and which can wear out, become clogged with particles, and provide an uneven flow profile across the channel [8,9]. The driving force in a nonmechanical micropump, on the other hand, can be based on nonmechanical energy (e.g., magnetics or electricity) that is converted into kinetic momentum. For the special case of pumping electrically conductive fluids (i.e., electrolytes), electrokinetic techniques are more effective. The application of electrokinetics in the development of microfluidic devices has attracted great attention over the past decades, owing to its fairly simple implementation and reliability due to no moving parts [10].

For relatively low electrically conductive fluids (i.e., <1 S/m), electroosmotics is the commonly used technique, which is based on the formation of an electric double layer in the bulk of the electrolyte next to the channel surfaces. The electroosmotic effect can become weak if the electric double layer thickness decreases due to the high electrical conductivity of the electrolyte. This causes the electroosmotic-based devices to become inefficient for manipulation of biological fluids (i.e., blood), since the electrical conductivity of biofluids is relatively high (i.e., ~1 S/m).

In contrast, the electrothermal technique can be used to pump high conductivity fluids [11,12,13,14,15,16,17,18]. When a temperature gradient is imposed in the bulk of a fluid by either Joule heating or an external heat source, due to the interaction of the electric field with the nonuniform electric permittivity and conductivity, electrothermal flow is generated [19,20]. Typically, a nonuniform AC electric field is imposed by a symmetric [12,21,22] or asymmetric [19,23,24] electrode pair, which can generate ACET force in the bulk of an electrolyte. The resultant fluid flow forms vortices on top of the electrodes, with the maximum velocity at the edges of the electrodes near the gap between the two electrodes in each pair. In order to improve ACET fluid flow, modifications such as multiphase actuation [25,26], DC-biased actuation [15,27,28], and adding external heat films [19,29] have also been suggested. In typical ACET devices, an array of microelectrodes is fabricated at the bottom of a microfluidic channel such that the resultant ACET flow can cause a net flow through the channel. Electrokinetic devices with different coplanar electrode geometries have been suggested for mixing analytes in microfluidics [30,31,32]. The application of multiarrays of microelectrode pairs has been shown to increase the net flow rate of an ACET device significantly [33]. Having electrode pairs on all sides of a microfluidic channel with circular and rectangular cross-sections has been suggested for mixing and pumping applications [33,34]. However, the fabrication of microelectrodes in those designs (especially on the perimeter of a channel with circular cross-section) is still a major challenge.

A cost-effective technique for making a double-array ACET device is by aligning two planar substrates with electrode arrays on top and bottom of a polydimethylsiloxane (PDMS) channel. Here, we numerically study an easy-to-fabricate double-array ACET device for the application of simultaneous pumping and mixing of biological samples. Each array consists of coplanar microelectrode pairs. The transversal mixing at a rectangular cross-section of the microfluidic channel with different coplanar electrode geometries placed on top and bottom is studied, and an optimal electrode design is identified. Then the longitudinal pumping achieved with electrodes placed at different orientations relative to the channel length are compared. By adjusting the electrode array orientations, such a double-array ACET device can be used for pure micropumping, pure micromixing, or simultaneous pumping and mixing. It should be noted that (as opposed to the study of Gao and Li [34]) our proposed design is easy to fabricate, contains two layers of microelectrode arrays, and can be used as a micropump, micromixer, or both (as opposed to the study of Salari et al. [33]) by simply adjusting the angle of the electrode array patterned on the substrates. The ease of substrate repositioning means this device is suitable for many fluid transport lab-on-a-chip applications.

## 2. Numerical Simulation

When an AC electric field is applied to an electrolyte solution, which has a high electrical conductivity, a temperature gradient is generated in the bulk of the solution due to the Joule heating phenomenon, and can be expressed as [33]:

(1) $$k\nabla^{2}T+\sigma {\left|E\right|}^{2}=\rho C_{p}\frac{\partial T}{\partial t}$$

where *T*, ***E***, *σ*, *k*, *ρ*, and *C_p_* are temperature, electric field, electrical conductivity, thermal conductivity, density, and specific heat (at constant pressure), respectively.

In a homogenous medium, the Laplace equation can be expressed as:

(2) $$\nabla^{2}V=0$$

where E=−∇V.

The charge conservation equation can be calculated as:

(3) $$\frac{\partial \rho_{c}}{\partial t}+\nabla \cdot \left(\sigma E\right)=0$$

where $\rho_{c}$ is the free charge density.

The resultant time-averaged ACET body force exerted on the liquid can be presented as:

(4) $$\langle F_{et}\rangle =-\left[\frac{1}{2}\left(\frac{\nabla \sigma }{\sigma }-\frac{\nabla \varepsilon }{\varepsilon }\right)E\times \frac{\varepsilon E}{1+{\left(\omega \tau \right)}^{2}}+\frac{1}{2}\nabla \varepsilon {\left|E\right|}^{2}\right]$$

where ε,  ω, and τ are permittivity, angular frequency, and charge relaxation time, respectively.

The dynamics of an incompressible fluid flow of low Reynolds number is governed by the Navier–Stokes and continuity equations:

(5) $$-\nabla p+\eta \nabla^{2}u+\langle F_{et}\rangle =0,\;\nabla \cdot u=0$$

where ***u***, *η*, and *p* are the velocity, dynamic viscosity, and pressure, respectively.

The species concentration field in a liquid is governed by the convection–diffusion equation as:

(6) $$\frac{\partial C}{\partial t}=\nabla \cdot \left(D\nabla C\right)-\nabla \cdot \left(uC\right)$$

where *C* and *D* are species concentration and diffusion coefficient, respectively.

The numerical approach followed in this manuscript is based on the scaling law simplification and low temperature rise assumption, and thus, the energy and electrical equations can be decoupled and changes to fluid properties can be assumed to be small [21,22,23,25,33,35]. Although not the focus of this article, it should be noted that for the cases where the temperature rise is significant, the electrothermal coupling cannot be neglected, and temperature-dependent effects (e.g., electrical conductivity) should be considered [36].

## 3. Results and Discussion

The configuration of microelectrodes on the channel walls of a microfluidic device is critical for the direction of the resultant fluid flow. If a coplanar electrode array is used in an ACET device, the axis of the resultant microvortices will be parallel to the electrode lengths [16,25,36]. If the orientation of the array is set such that microelectrodes are placed parallel to the microfluidic channel, then the ACET microvortices will be mostly dragging the liquid on the cross-sectional plane of the channel. In such orientation of microelectrodes, no net flow along the channel can be generated, rather, a circular motion capable of mixing the liquid transversally will be achieved (i.e., a pure micromixing strategy). If the microelectrode pairs are rotated 90°, such that they become perpendicular to the channel length, then the resultant ACET microvortices will only drag the liquid along the channel, and a net flow will be generated (i.e., pure micropumping strategy). In order to characterize ACET transverse flow generated by coplanar microelectrodes placed along a microchannel, we model a cross-section (i.e., 300 × 300 μm) of an ACET microfluidic device consisting of two commonly used array configurations (i.e., symmetric and asymmetric) fabricated on glass substrates and separated by a PDMS microfluidic channel. The details of our modeling approach using COMSOL Multiphysics (V5.3, Burlington, MA, USA), as well as its experimental validation, have been reported previously [33,37]. Briefly, we first solve the electric field for the channel domain assuming zero polarizability on the channel walls except for the areas where electrodes are placed. Then, we model the heat transfer in the entire device (including the channel domain and the wall/substrate domain) assuming Joule heating as a heat source in the channel domain, and ambient temperature on the outer surfaces of the device. Then, the fluid dynamics inside the channel domain is modeled assuming no-slip condition on the channel wall, and ACET body force (Equation (4)) as the external force acting on the liquid. At the end, the convection–diffusion equation is solved to obtain the species concentration distribution throughout the channel domain.

**Figure 1:**
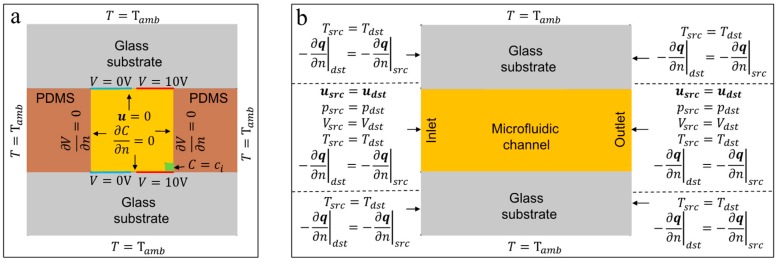
The boundary conditions used for different domains (i.e., electrical, thermal, laminar flow, and species transport) in the 2D modeling of the ACET device. (**a**) shows the cross-sectional view of the device. (**b**) shows the side view of the device. The parameters q and $T_{amb}$ are the heat flux and ambient temperature, respectively. The periodic quantities are referred to using *src* and *dst* subscripts to represent *source* and *destination*, respectively. The schematics are not drawn to scale. Note that the electrode dimensions (red and blue) and the species release area (green) in (**a**) are shown as an example.

Table 1 shows the design parameters and fluid properties used in our numerical simulations. Schematics of the boundary conditions used in our multiphysics approach are demonstrated in Figure 1.

**Figure 2:**
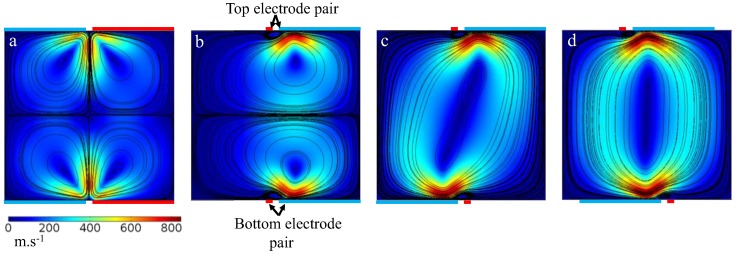
The magnitude of 2D velocity field and flow streamlines are shown in a cross-section of an ACET microfluidic device consisting of longitudinal microelectrodes parallel to the microfluidic channel (coming out of the page vertically). The top and bottom substrates (not shown) contain two (**a**) symmetrical (Design I) and asymmetrical (i.e., (**b**) Design II and (**c**) Design III) microelectrode pairs in contact with the fluid. The electrodes in (**a**) have equal 140 μm widths, while in (**b**–**d**) the wide electrode is 120 μm and the thin one is 20 μm. The gap width between electrodes for all cases is 20 μm. Actuated (red) and ground (blue) electrodes are as indicated. As shown, regions of strongest fluid flow are near the gap between the two electrodes. Four and two symmetrical microvortices are generated due to the application of Design I and Design II, respectively, whereas, in Design III, the entire cross-section of the channel is swept by only one set of microvortices. If the electrode pairs in (**c**) are repositioned 20 μm horizontally towards the center (as shown in (**d**)), the resultant streamlines become more circular, sweeping the entire cross-section more uniformly.

As shown in Figure 2, we model one symmetric pair (Design I) with dimensions of 140/20/140 μm (i.e., electrode width/gap/electrode width, respectively), and two identical asymmetric pairs (Designs II and III) with dimensions of 20/20/120 μm. Considering the fact that the direction of ACET flow is reversed if the electrode placement is switched, we chose two different asymmetrical designs. In Design I, the two pairs on top and bottom of the channel are placed with identical orientation, whereas, in Design II, the placement of the electrodes is switched such that the direction of the ACET flows generated by the two pairs match. As shown in Figure 2a, the streamlines formed in a symmetrical microelectrode configuration consist of four identical sets of microvortices, each of which sweeps roughly one quarter of the channel cross-section. When asymmetrical electrodes are used instead, the number of microvortices sets are reduced to two (Figure 2b) or one (Figure 2c) depending on the orientation of the microelectrodes. In all three designs, the maximum velocity appears to occur near the gap and above the edges of the electrodes with a larger region of maximum velocity for the asymmetrical designs. If the asymmetrical electrode pairs in Design III are moved 20 μm horizontally towards the center, the circular flow streamlines will become vertically symmetric, which can sweep all corners of the cross-sectional surface uniformly (Figure 2d). This means that regardless of where the analytes are released on the cross-sectional area of the channel, the transport of analytes in Design III and Design III-optimized (Figure 2c,d) will be mainly limited by convection, as streamlines sweep a larger area, whereas in Designs II and I, the maximum sweep area of the streamlines is half and a quarter of the channel cross-section, respectively. 

**Figure 3:**
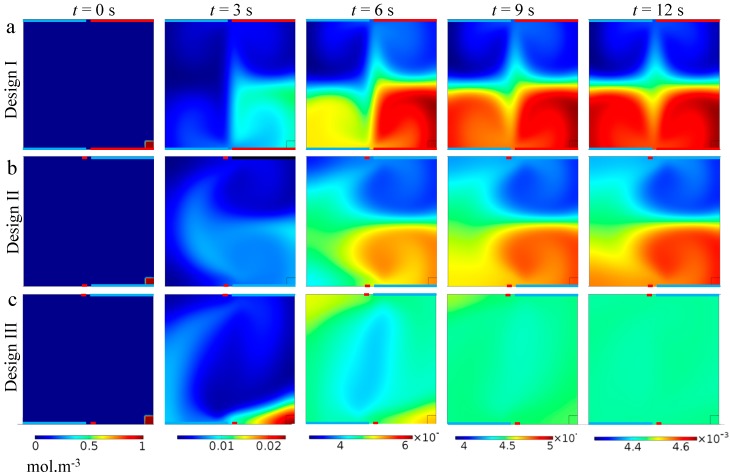
Concentration distribution across the channel cross-section of the ACET microfluidic device over time for the three designs. The electrodes in (**a**) have equal 140 μm widths, while in (**b**,**c**), the wide electrode is 120 μm and thin one is 20 μm. The gap distance between electrodes for all cases is 20 μm. An analyte with an initial concentration of 1 mol·m^−3^ is introduced in a 20 × 20 μm region at the lower right corner of surface at *t* = 0 (small red square). A rapid mixing within each set of microvortices is observed due to strong convection in those areas, however, analyte transfer across the regions swept by separate sets is largely limited by diffusion. As shown, a uniform distribution of analyte is achieved in the shortest time (*t* = 12 s) when Design III is used.

To better visualize the transverse fluid mixing and to compare the designs, we introduce analytes to a small region (i.e., 20 × 20 μm) at one corner of the channel cross-section and monitor the concentration distribution over time (Figure 3). This way we can visualize the convective flux across the cross-sectional area of the ACET device. As shown, the analyte concentration is convected within each set of microvortices rapidly. However, analyte transfer across every two sets of microvortices is mainly limited by diffusion at the boundaries, which occurs in a much shorter time-scale compared to convection. This limitation has the largest impact on analyte transfer in Design I, where there are four sets of microvortices. In contrast, in Design III, convection is the main transport phenomenon across the entire surface, yielding an almost uniform concentration distribution at *t* = 12 s.

**Figure 4:**
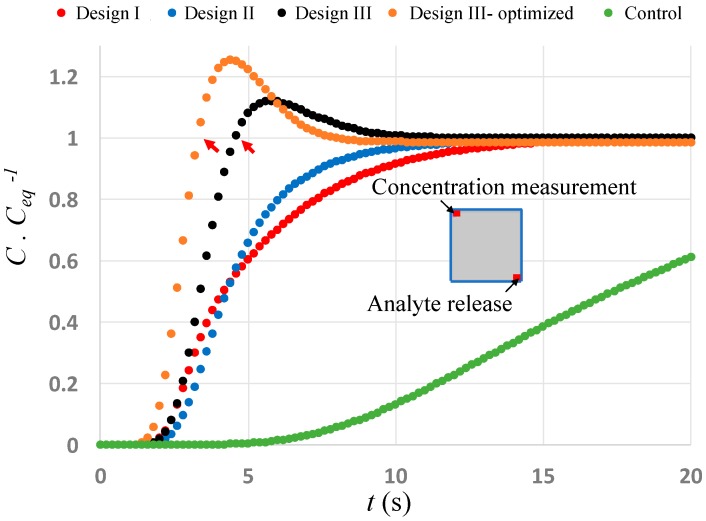
Average changes of concentration ratio inside a 20 × 20 μm region located diagonally across from the initial analyte release region. The inset image shows the location of analyte release and analyte measurement on the channel cross-section. $C_{eq}$ represents equilibrium concentration. The fastest increase in concentration is for the optimized Design III (orange line), where a higher than equilibrium concentration is achieved in less than 3.4 s. Since convective fluxes are critical in analyte transport, the mixing in all of the designs is significantly higher than a diffusion-only mixing strategy (green line), where, as in most microfluidic applications, the fluid is not actively mixed transversally while passing through a microfluidic channel. The diffusion-only control curve (green) reaches equilibrium ($C\cdot C_{eq}^{-1}=1$ ) at t = ~ 70 s (not shown).

We also measure the average concentration ratio inside a region with dimensions equal to the analyte introduction’s region and located diagonally across from it (Figure 4). As expected, the concentration increases rapidly in Design III due to the strong convective flux and surpasses the final equilibrium concentration within only 4.6 s (and 3.4 s for the optimized Design III) (shown by red arrows) after which it decays gradually, due to diffusive flux, to equilibrium concentration. This rapid increase in concentration is mainly due to the high analyte convection happening as a result of circulating flow streamlines. The graphs representing Design I and II, however, show a slower increase in concentration, due to the comparable magnitudes of convective and diffusive fluxes, which eventually reach equilibrium at *t* = 18 s. When no ACET flow is generated (Figure 4—control, green line), diffusion is the only mechanism of analyte transport (unless a net flow is imposed externally), which takes 74% longer time for the concentration graph to reach the equilibrium concentration at *t* = ~70 s (not shown on the graph). The results show that by adjusting the applied voltage pattern and position of the asymmetrical microelectrodes in the two arrays, due to the resultant circular flow streamlines, the time it takes for the analytes to be convected across the channel cross-section is reduced by 95% compared to the diffusion-based transport regime (Figure 4—control, green line), and by 80% compared to a conventional two-layer ACET device (Figure 4—Designs I and II, red and blue lines).

**Figure 5:**
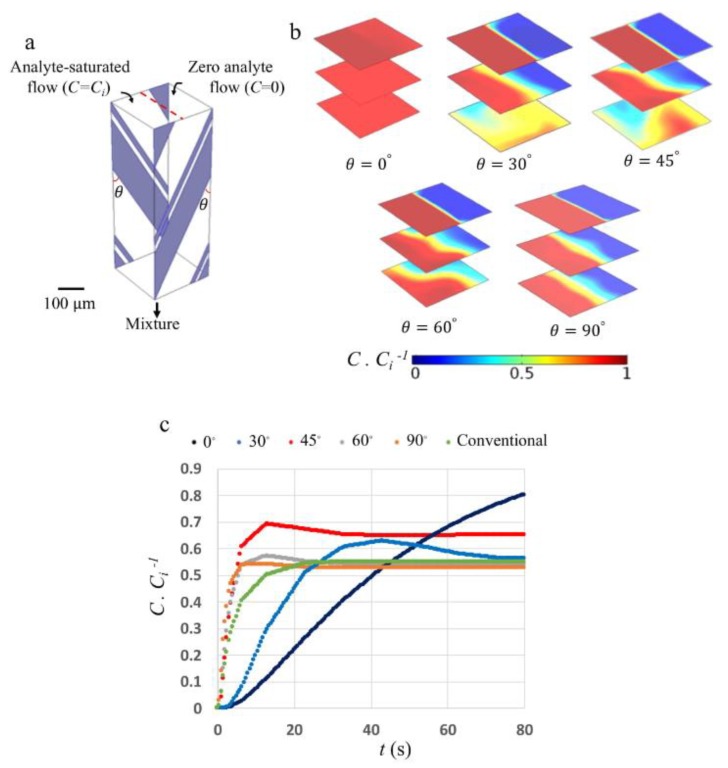
(**a**) shows the geometry of the microfluidic channel with the microelectrodes placed at an angle of θ. Two flows, one of which has an analyte concentration of *C_i_* = 10^−7^ mol·m^−3^, are mixed and pumped due to the ACET effect. To save computational time, only a portion of the microfluidic channel, covering one electrode pair with periodic boundary conditions at the two ends (i.e., inlet and outlet) of the channel, is modeled. (**b**) shows the steady-state concentration distribution at three different cross-sectional surfaces separated by an equal distance of 150 μm for 5 different microelectrode angles. At $\theta =0^{{}^{\circ}}$, the device becomes a pure micromixer, whereas, at $\theta =90^{{}^{\circ}}$, the device acts as a micropump. (**c**) shows the outlet dimensionless concentration versus time. For $\theta \geq 45^{{}^{\circ}}$ (red, grey, and orange lines), a rapid increase in concentration occurs within 8 s, whereas, for $\theta <45^{{}^{\circ}}$ (blue and dark blue lines), concentration changes occur over a longer period of time. The results of a conventional single-array ACET device, which typically has an electrode angle of $\theta =90^{{}^{\circ}}$ (green line), are included for comparison.

For the rest of this work, we utilize the Design III electrode configuration to study a simultaneous pumping and mixing strategy in a typical double-array ACET microfluidic device. We introduce two side flows into the microfluidic channel with identical cross-sectional dimensions as modeled above. Since, in a 3D configuration, the two microelectrode arrays can be placed at different angles relative to the channel length, we model one pair of electrodes separated by 140 μm and located at 0°, 30°, 45°, 60°, and 90° (Figure 5a) in order to study both pumping and mixing phenomena when the analyte is released at one inlet only. Figure 5b shows the analyte concentration at different cross-sections along the microfluidic channel after a relatively long time, so that a steady state is reached. As shown, for electrode angle of θ=0°, the microfluidic device becomes a pure mixer, capable of mixing analytes transversally, while transport along the channel mainly occurs by diffusion. Increasing the angle to θ=45° causes the average concentration to reach $∼0.5\;\mathrm{mol}\cdot m^{-3}$ near the outlet. In designs with larger angles (θ>45°), the generated transverse flow becomes weaker than the longitudinal flow, with diffusion becoming the major transport mechanism across the boundary of the two flows. As depicted in Figure 5c, for θ≥45°, average concentration ratio at the outlet increases rapidly, while for θ<45°, the concentration increases ~200% slower. A conventional ACET device consisting of a single array of microelectrode pairs at an angle of θ=90° is also modeled and the results are shown in Figure 5c. Comparing ACET devices with different electrode orientations reveals that, at angle θ=45°, a higher steady-state concentration can be achieved in a shorter time. Figure 5c shows that changing the angle of the electrode pairs affects the pumping flow rate such that designs with different electrode orientations reach different steady-state concentrations at the outlet. At θ=90°, the ACET device generates the highest pumping flow rate, which causes the steady-state concentration to reach as low as ∼0.52 in 10 s, whereas, at θ=0°, the highest mixing flow rate is generated, which causes the steady-state concentration to approach unity 900% slower. The highest outlet concentration achieved in the shortest time is for a device with θ=45°, where the steady-state concentration of ∼0.65 is achieved in ∼25 s.

Our results also show that the average ratio of transversal to longitudinal convective fluxes are higher for the designs of θ=45° and 30°. For example, this ratio, at a cross-section 300 μm away from the inlet, is ∼2.3 and ∼0.7 for the designs of θ=30° and 60°, respectively.

In order for a double-array ACET microfluidic device to be used primarily for pumping liquids with the minimum mixing, designs with the minimal ratio of transversal to longitudinal convective fluxes need to be utilized. This includes designs with electrode angles close to θ=90°. In contrast, for applications where simultaneous pumping and mixing are desirable, designs with the highest ratio of transversal to longitudinal convective fluxes are preferable. This includes designs with electrode angles within the range of, or very close to, $30^{{}^{\circ}}\leq \theta \leq 45^{{}^{\circ}}$. The extent of mixing can be further optimized by adjusting the microelectrode angles based on the channel geometry and electrode pair dimensions.

## 4. Conclusions

In conclusion, we have designed and modeled an easy-to-fabricate double-array ACET device which can be used for high conductivity biofluid applications. Depending on the orientation of the electrode arrays to each other on the top and bottom of the channel, different mixing, pumping, and simultaneous mixing and pumping regimes can be achieved. One way of fabricating such an ACET device is to micromachine microfluidic channels in a mold, which is then used for casting a PDMS gasket. Two glass slides covered by interdigitated microelectrode arrays can then be aligned on top and bottom of the gasket. The whole device can be clamped later to avoid leakage [33]. Having the microelectrode arrays on the top and bottom of a microfluidic channel enables the versatility of the array orientation relative to each other in order to precisely control the degree of mixing when the fluid is pumped in the channel, as opposed to having a spiral microelectrode pair in a cylindrical device, where changing the mixing-to-pumping ratio requires the fabrication of a new device [34]. One fabrication challenge of our design could be the alignment of the two microelectrode arrays before sandwiching a PDMS gasket in between, in order to achieve the optimum mixing and pumping output. Continuing this work, we hypothesize that an array of asymmetrical microelectrodes can be designed such that in every two successive pairs, one contributes mostly to transversal flux, while the other one contributes to longitudinal flux. An ACET device consisting of a single-array or double-array electrode pairs with this configuration can also fulfill a simultaneous mixing and pumping strategy which can be further investigated in the future.

## Figures and Tables

**Table 1:** Design parameters and fluid properties used in the numerical simulation.

Property	Value
Substrate thermal conductivity (W/m·K)	1.1
PDMS thermal conductivity (W/m·K)	0.16
Ambient temperature, $T_{amb}$ (K)	293.15
Channel cross-section (μm)	300 × 300
Wide electrode width (μm)	120
Narrow electrode width (μm)	20
Gap between electrodes in each pair (μm)	20
Actuation frequency (kHz)	100
Actuation voltage $\left(V_{\mathrm{rms}}\right)$	10
Fluid conductivity (S/m)	0.225
Diffusion coefficient, D $\left(m^{2}/s\right)$	$10^{-9}$
Inlet concentration—3D model $\left(\mathrm{mol}/m^{3}\right)$	$10^{-7}$
Initial concentration at the corner—2D model, $c_{i}$ $\left(\mathrm{mol}/m^{3}\right)$	1
